# Supplementary material for: A Broad-Spectrum Chemokine Inhibitor Blocks Inflammation-Induced Myometrial Myocyte–Macrophage Crosstalk and Myometrial Contraction
Source: Cells. 2021 Dec 31;11(1):128. doi: 10.3390/cells11010128 (PMC8750067; doi:10.3390/cells11010128)
Supplement: Supplementary file 1 [file cells-11-00128-s001.zip › cells-1520741-supplementary.pdf]

Figure S1 – Supplemental (Boros et al.)

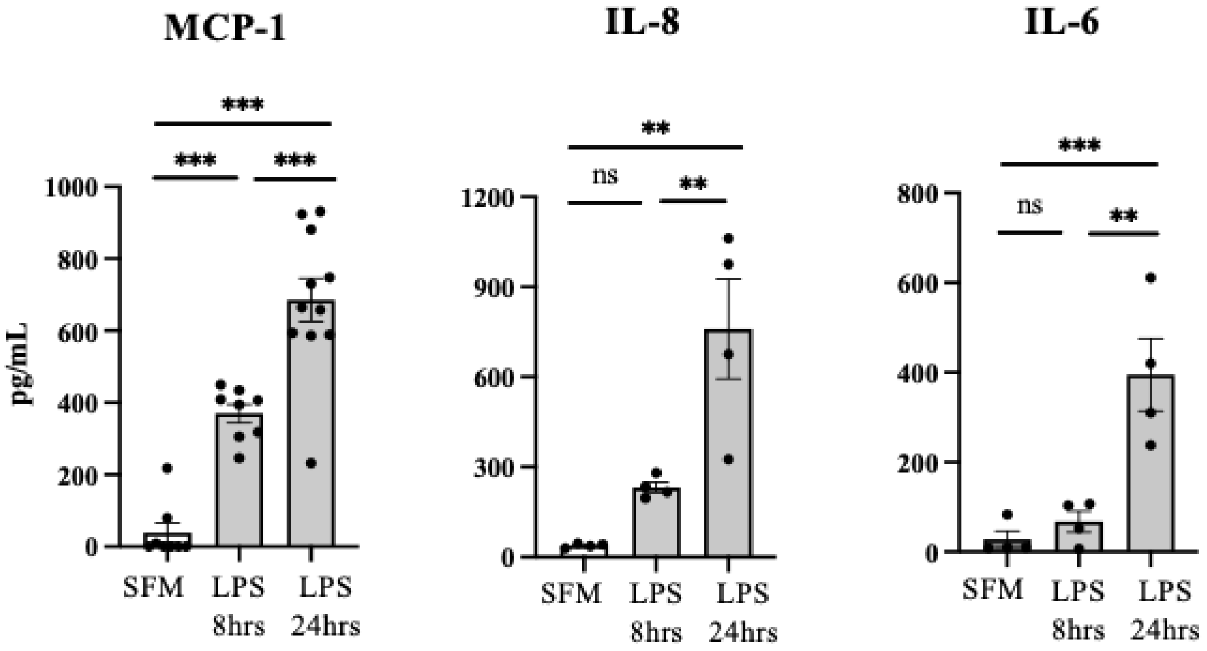

Suppl Figure S1: Temporal profile of infection-induced cytokine and chemokine secretion by human myometrial cells. Human myocytes isolated from myometrial biopsies of term pregnant women undergoing elective caesarean sections (n=4-11) were treated with LPS (100 ng/ml), or vehicle (serum-free media, SFM) for 8 or 24 hours. Cell culture media conditioned by primary myometrial cells were collected and analyzed by specific ELISAs. Concentrations of secreted MCP-1, IL-8, and IL-6 detected are shown in pg/ml. Dots represent individual cell lines, (n=4-11). Data are presented as mean ± SD. Statistical significance was determined through One-Way ANOVA followed by Dunnette's multiple comparisons test. '**' denotes statistical significance at P<0.01, and '***' at P<0.001.

Figure S2 – Supplemental (Boros et al.)

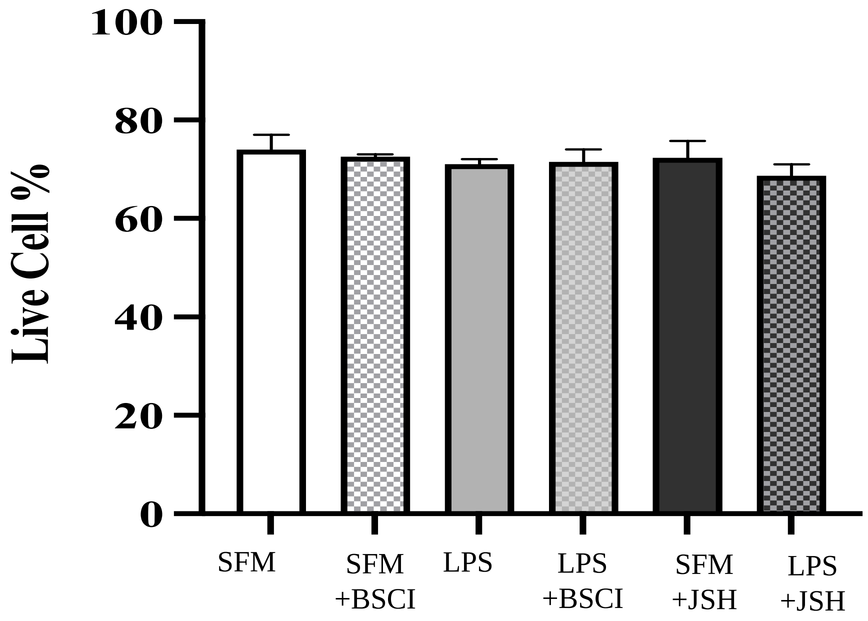

Suppl Figure S2: Viability of collagen-embedded human myocytes. After treatments myocytes from collagen gels were released via 0.1% collagenase digestion. Cells were collected by centrifugation and viability was measured using an automated cell counter instrument with Trypan blue viability dye. Data are presented as Mean ± SD. Statistical significance was determined through One-Way ANOVA followed by Dunnette's multiple comparisons test.
